# Natural Deep Eutectic Solvents Based on Choline Chloride and Phenolic Compounds as Efficient Bioadhesives and Corrosion Protectors

**DOI:** 10.1021/acssuschemeng.2c01976

**Published:** 2022-06-13

**Authors:** Matías L. Picchio, Daniela Minudri, Daniele Mantione, Miryam Criado-Gonzalez, Gregorio Guzmán-González, Ruth Schmarsow, Alejandro J. Müller, Liliana C. Tomé, Roque J. Minari, David Mecerreyes

**Affiliations:** † Instituto de Desarrollo Tecnológico para la Industria Química (INTEC), CONICET, Güemes 3450, Santa Fe 3000, Argentina; ‡ Departamento de Química Orgánica, Facultad de Ciencias Químicas (Universidad Nacional de Córdoba), IPQA−CONICET, Haya de la Torre y Medina Allende, Córdoba 5000, Argentina; § POLYMAT and Department of Polymers and Advanced Materials, Physics, Chemistry and Technology, Faculty of Chemistry, University of the Basque Country UPV/EHU, Paseo Manuel de Lardizábal, 3, 20018 Donostia-San Sebastián, Spain; ∥ POLYKEY Polymers, Joxe Mari Korta Center, Avda. Tolosa 72, 20018 Donostia-San Sebastian, Spain; ⊥ IKERBASQUE, Basque Foundation for Science, Plaza Euskadi 5, 48009 Bilbao, Spain; # LAQV-REQUIMTE, Department of Chemistry, NOVA School of Science and Technology, FCT NOVA, Universidade Nova de Lisboa, 2829-516 Caparica, Portugal

**Keywords:** Polyphenols, Eutectic mixtures, Catechol chemistry, Metal−ligand coordination, Tissue adhesives, Corrosion inhibitors

## Abstract

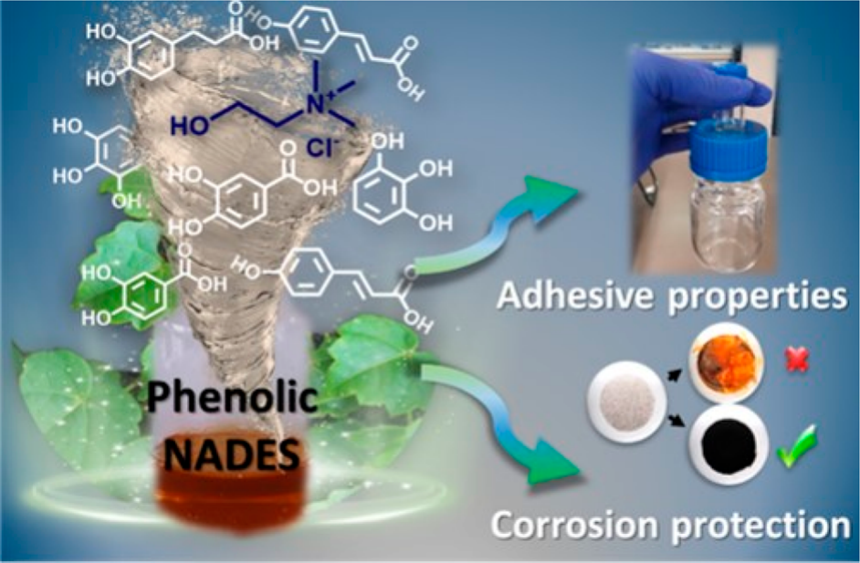

Natural deep eutectics solvents (NADES), owing to their high solvation capacity and nontoxicity, are actively being sought for many technological applications. Herein, we report a series of novel NADES based on choline chloride and plant-derived polyphenols. Most of the obtained phenolic NADES have a wide liquid range and high thermal stability above 150 °C. Among them, small-sized polyphenols, like pyrogallol, vanillyl alcohol, or gentisic acid, lead to low-viscosity liquids with ionic conductivities in the order of 10^–3^ S cm^–1^ at room temperature. Interestingly, polyphenols possess valuable properties as therapeutic agents, antioxidants, adhesives, or redox-active compounds, among others. Thus, we evaluated the potential of these novel NADES for two applications: bioadhesives and corrosion protection. The mixture of choline chloride-vanillyl alcohol (2:3 mol ratio) and gelatin resulted in a highly adhesive viscoelastic liquid (adhesive stress ≈ 135 kPa), affording shear thinning behavior. Furthermore, choline chloride-tannic acid (20:1) showed an extraordinary ability to coordinate iron ions, reaching excellent corrosion inhibitive efficiencies in mild steel protection.

## Abstract 2

A series of natural polyphenols are deeply examined as novel hydrogen bond donors for preparing functional natural deep eutectic solvents (NADES).

## Introduction

Deep eutectic solvents (DES) have emerged as a new class of mixtures of pure compounds for which the eutectic point temperature is far below that of an ideal liquid mixture.^1,2^ The established opinions hold that the primary driving forces for DES formation are hydrogen bonding or ionic interactions between a hydrogen-bond donor (HBD) and a hydrogen-bond acceptor (HBA).^3^ Many of the DES general properties are similar to those of ionic liquids (ILs), including low volatility, high thermal stability, and good ionic conductivity. However, whereas the ILs’ green characters have often been questioned due to their nondegradabilities, high toxicities, and sustainabilities, DES are typically biodegradable, nontoxic, inexpensive, and simpler to prepare.^4^

These intriguing mixtures were first reported by Abbot et al.^5^ in 2003, who observed an abnormal deviation in the ideal melting temperature of the choline chloride (ChCl)/urea combination (1:2 mole fraction). Since then, the field has been expanded broadly to incorporate many novels DES, and a library of potential constituents has been identified, allowing the design of a plethora of new solvents for technological applications.^6,7^ On the podium of these strides appear natural deep eutectic solvents (NADES), a particular type of DES where their constituents are primary metabolites or natural compounds.^8,9^ The most common NADES are based on mixtures of ChCl with organic acids,^10,11^ polyalcohols,^12,13^ and sugars^14,15^ or a combination of these natural molecules with amino acids.^16−18^ However, most traditional NADES lack functionalities, and new green mixtures are urgently demanded to expand the applicability fields of these natural solvents. In this sense, phenolics and flavonoids, nearly ubiquitous molecules found in vegetables and fruits, have been barely investigated for this purpose. Many studies have focused on the potentials of NADES to extract polyphenols from biomass sources,^19−21^ but the abilities of these natural compounds to form eutectic mixtures remain almost unexplored.^22^

Since these bioderived molecules show valuable properties as antioxidants, anti-inflammatorys, anticancers, and antibacterials, phenolic NADES could open new avenues in the emerging field of therapeutic deep eutectic solvents (THEDES).^23,24^ On the other hand, catechol and pyrogallol-bearing NADES particularly would benefit from functionalities, redox-active properties, and metal–ligand coordination abilities that could also be exploited to create new innovative functional materials for electrochemical devices,^25^ water remediation,^26^ underwater adhesives,^27^ and so on.^28^

**Figure 1:**
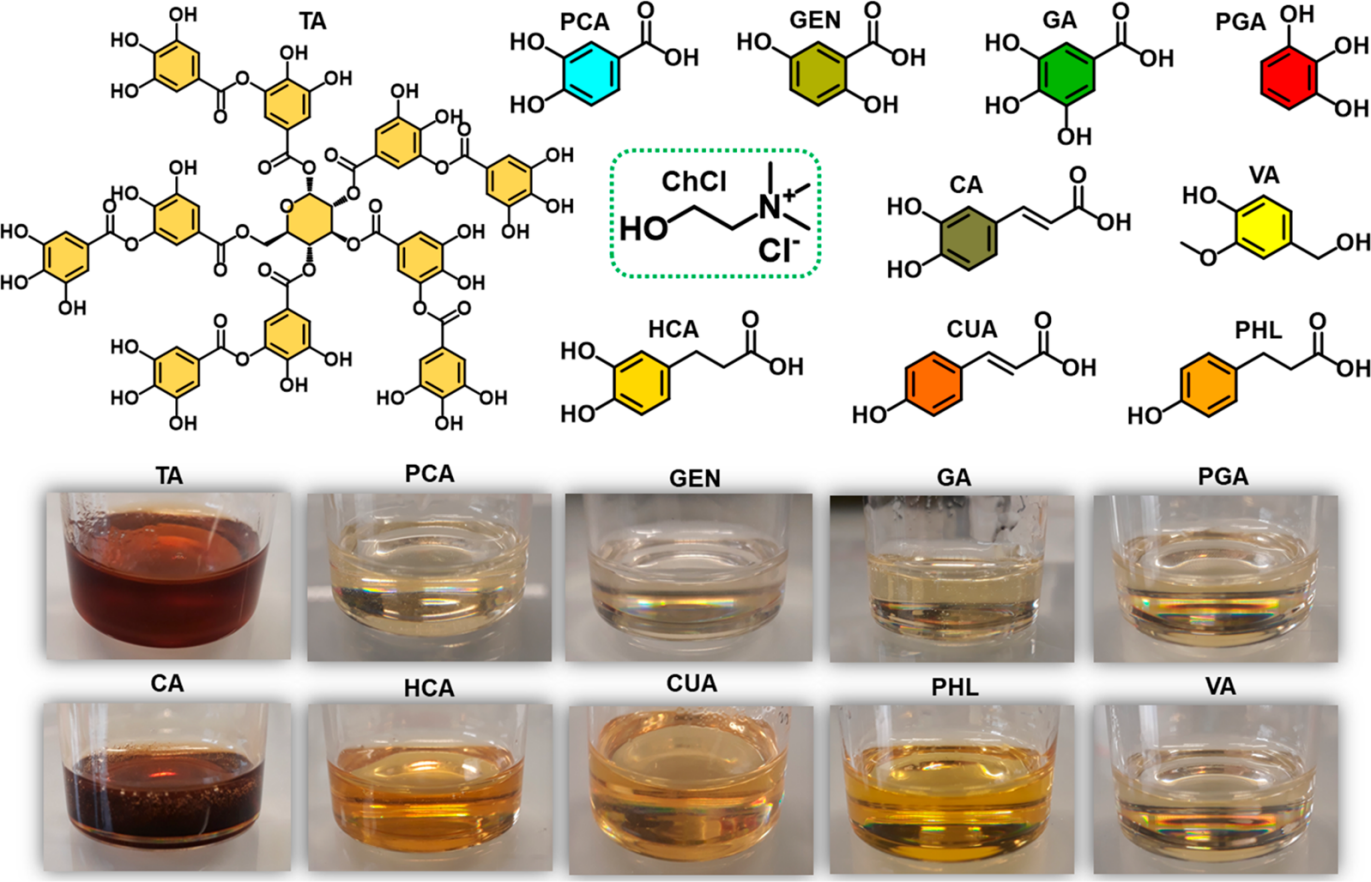
Chemical structures of ChCl and phenolic compounds used to prepare the different NADES illustrated in the pictures.

In this letter, we present a new family of NADES based on ChCl and a variety of phenolic compounds, including tannic acid (TA), protocatechuic acid (PCA), gentisic acid (GEN), gallic acid (GA), pyrogallol (PGA), caffeic acid (CA), hydrocaffeic acid (HCA), p-coumaric acid (CUA), phloretic acid (PHL), vanillyl alcohol (VA), and quercetin (QUE). The chemical structures of the compounds used and images of the obtained NADES are shown in Figure 1 and Figure S1 of the Supporting Information (SI). The novel prepared NADES were evaluated in terms of their thermal stabilities, ionic conductivities, and rheological behaviors. As a proof of concept, ChCl-VA/gelatin materials and ChCl-TA coatings were also fabricated in order to evaluate the potentials of the proposed NADES as bioadhesives and corrosion protectors, respectively.

## Results and Discussion

The polyphenols-based NADES were prepared by the standard heating method. The solids HBA and HBD were first mixed and then heated at 95 °C until a clear liquid was formed. Different mixtures with defined stoichiometric proportions of HBD and HBA (typically, 2:1, 1:1, 1:2) were tested, and Table 1 summarizes those combinations that resulted in liquids at room temperature. Note that ellagic acid, vanillic acid (VAAc), and amino acid 3,4-dihydroxy-l-phenylalanine (L-DOPA) did not yield liquid mixtures with ChCl even at different molar ratios. On the other hand, some polyphenols, such as PCA, GA, CUA, and QUE, crystallized at room temperature (see footnotes of Table 1).

**Table 1:** Summary of Different NADES Studied in This Work Using ChCl as HBA and Natural Phenolic Compounds as HBD

HBD	Common plant source^a^	HBD water solubility 25 °C (mg mL^–1^)	ChCl:HBD molar ratio	Aspect
Tannic acid	Oak	2850	20:1	Highly viscous brownish liquid
Protocatechuic acid^b^	Plums	12.4	2:1	Viscous, yellowish liquid
Gentisic acid^c^	Christmas bush	12.3	2:1	Viscous transparent liquid
Gallic acid^d^	Tea leaves	11.9	3:1	Viscous transparent liquid
Pyrogallol	Eurasian watermilfoil	625	1:1	Transparent liquid
Caffeic acid	Coffee bean	<1	2:1	Highly viscous brownish liquid
Hydrocaffeic acid	Coffee bean	428	2:1	Low-viscosity yellowish liquid
p-Coumaric acid^e^	Asparagus officinalis	1.02	2:1	Orange liquid
Phloretic acid	Apple tree leaves	2.71	1:1	Orange viscous liquid
Vanillyl alcohol	Vanilla bean	2	2:3	Yellowish liquid
Quercetin^b^	Capers	2.63 × 10^–3^	4:1	Dark orange viscous liquid
Ellagic acid	Oak	0.82	2:1, 1:1, 1:2	NADES not formed
Vanillic acid	Vanilla bean	5.7	2:1, 1:1, 1:2	NADES not formed
L-Dopa	Velvet beans	3.3	2:1, 1:1, 1:2	NADES not formed

a Not intended to be an exhaustive list of all known natural sources.

b Changed to solid after 1 day.

c Changed to a highly viscous shimmering liquid after 1 day.

d Crystallize slowly at RT after a few hours.

e Changed to solid after 5 days.

Many NADES based on ChCl and organic acids are prone to degrade due to an esterification reaction between carboxylic groups and the alcohol moiety of the ammonium salt.^29^ Therefore, ^1^H NMR spectroscopy was performed to check the purities and stabilities of the polyphenols-based solvents. For all the NADES investigated, no evidence of ester formation in the range of 3.5–3.8 ppm was observed, indicating excellent stability (Figure S2).

**Figure 2:**
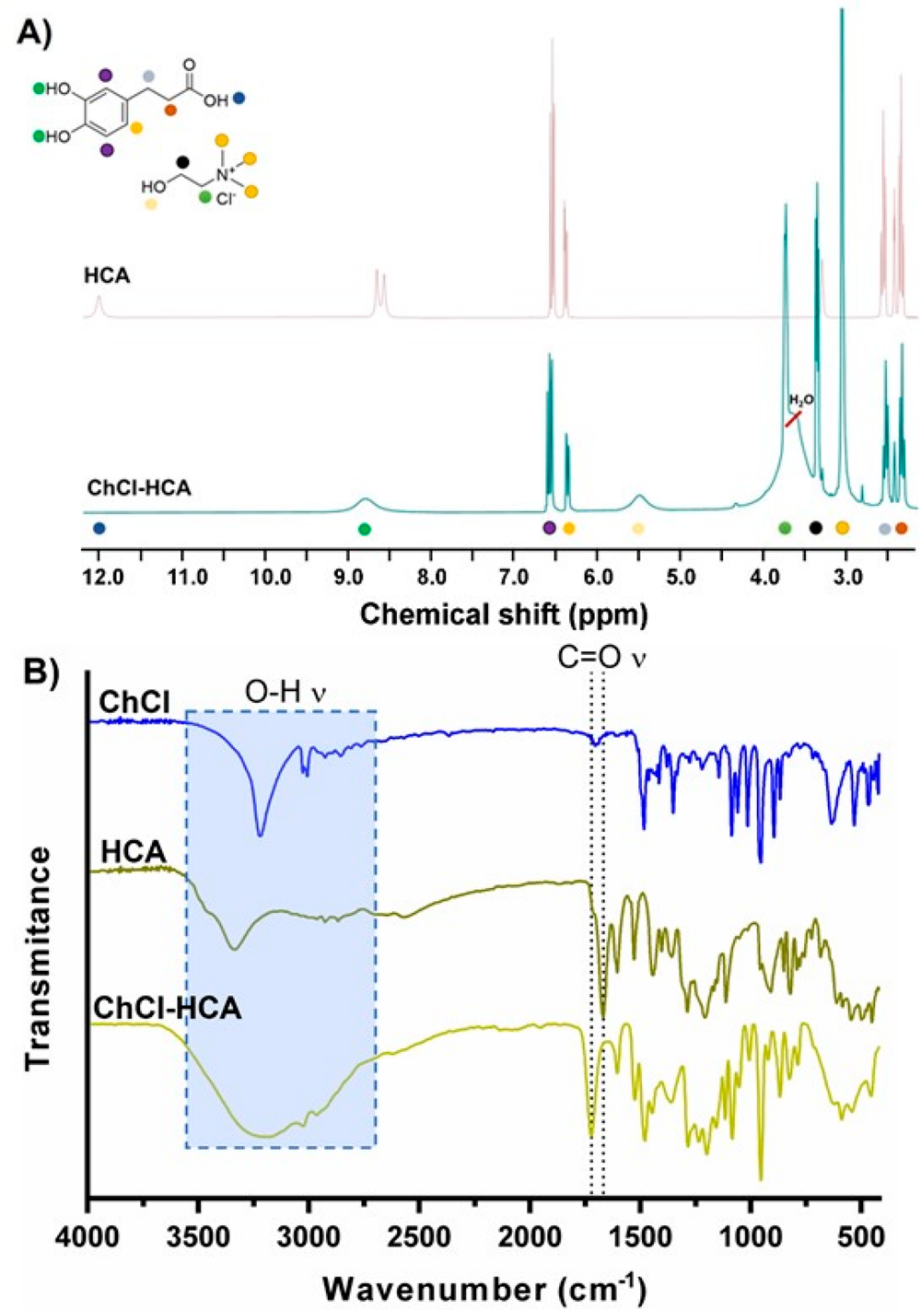
H^1^ NMR (A) and FTIR (B) spectra for pure components and ChCl-HCA NADES prepared by the heating method.

Besides, ^1^H NMR analysis revealed evident changes in the chemical signals of the NADES in comparison to the pure components, presumably due to strong interactions that drive the solvent formation. As an example, the ^1^H NMR spectra of ChCl-HCA and HCA are shown in Figure 2A. Note that the doublet at 8.5–8.75 ppm corresponding to the phenolic alcohols in HCA shifted and became a singlet after NADES formation. Further evidence of the strong interactions between ChCl and polyphenols was observed by FTIR analysis. As shown in Figure 2B, a shift in the carbonyl stretching peak (C=O ν) of HCA from 1666 to 1720 cm^–1^ and a band broadening in the region from 3500 to 2750 cm^–1^ (O–H ν) is noticeable in the spectra of the NADES (ChCl-HCA).

**Figure 3:**
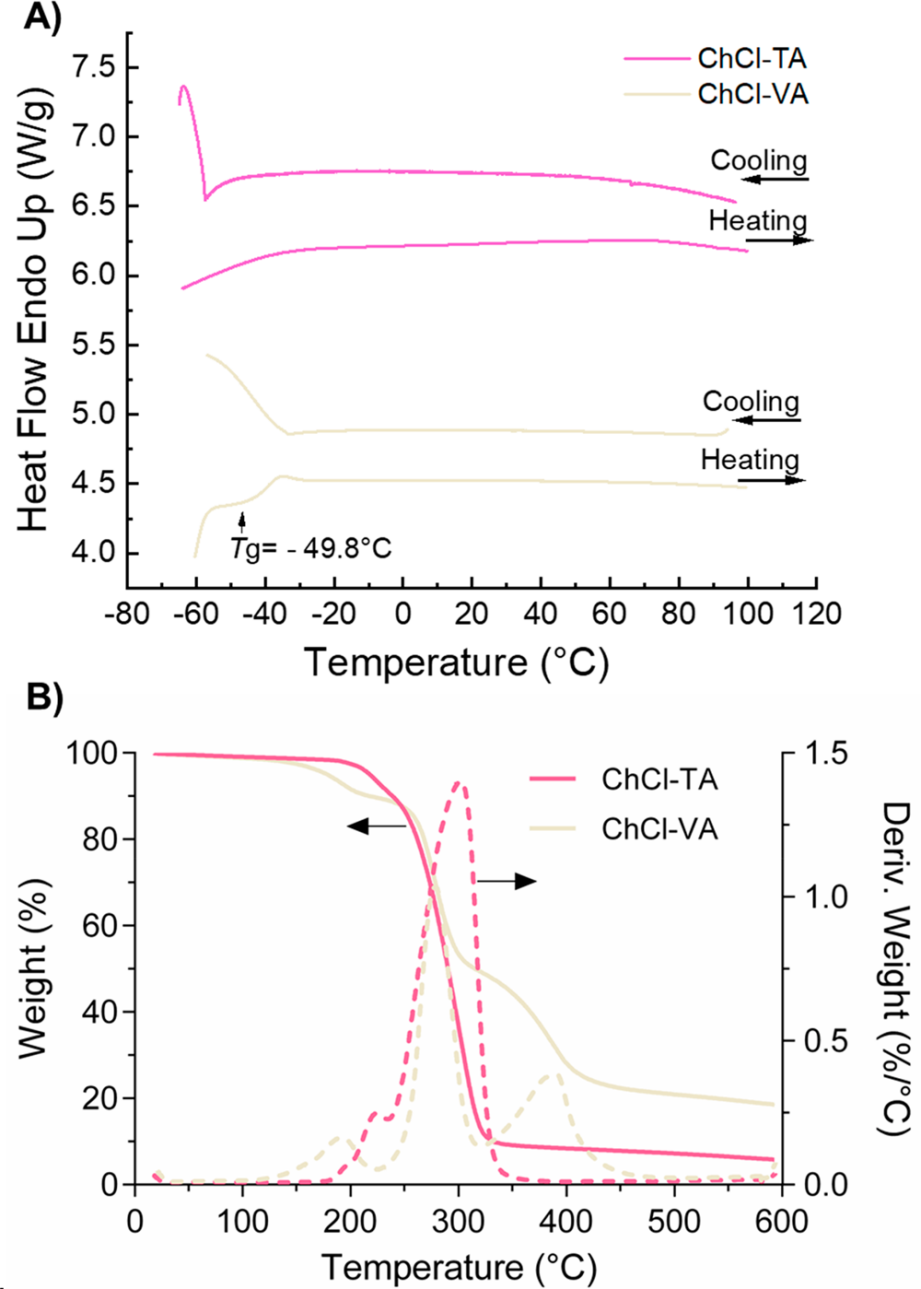
(A) DSC scans upon heating–cooling cycles for ChCl-TA and ChCl-VA. (B) TGA curve for ChCl-TA and ChCl-VA, including weight loss (solid lines) and derivative weight loss (dashed lines).

Table 2 summarizes the thermal properties of the NADES based on phenolic compounds. Except for the cases of ChCl-PCA, ChCl-GA, ChCl-CUA, and ChCl-QUE, which showed melting temperatures (*T*_m_) around 45–50 °C, all the other NADES studied exhibited a wide liquid range, and no phase transitions were detected in the temperature range studied (down to −60 °C). Only in the case of ChCl-VA was a glass transition temperature (*T*_g_) observed at −49.8 °C without a *T*_m_. In the case of the other liquid samples, it is presumed that their phase transition temperatures are below our DSC measuring temperature range. Furthermore, most NADES showed excellent thermal stabilities with decomposition temperatures at 5% (*T*_d5%_) and 50% (*T*_d50%_) of weight loss in the ranges of 117–226 °C and 259–315 °C, respectively. The lowest value of the maximum decomposition temperature (*T*_dmax_) was found for ChCl-GEN, while the highest was obtained for ChCl-TA. The DSC and TGA curves of two representative samples, ChCl-VA and ChCl-TA, are shown in Figure 3

**Table 2:** Thermal Properties of the Polyphenols-Based NADES^a^

NADES	*T*_g_ (°C)	*T*_m_ (°C)	*T*_d5%_ (°C)	*T*_d50%_ (°C)	*T*_dmax_ (°C)
ChCl-TA	N.O.	< −60	218.0	290.2	301.7
ChCl-PCA	N.O.	45.1	206.7	277.2	286.0
ChCl-GEN	N.O.	< −60	117.4	258.5	276.5
ChCl-GA	N.O.	42.1	164.8	272.9	288.7
ChCl-PGA	N.O.	< −60	215.1	289.3	301.1
ChCl-CA	N.O.	< −60	156.9	288.6	286.0
ChCl-HCA	N.O.	< −60	226.0	283.0	284.9
ChCl-CUA	N.O.	53.7	149.2	281.7	290.6
ChCl-PHL	N.O.	< −60	198.6	263.1	273.6
ChCl-VA	–49.8 °C	N.O.	178.6	315.5	278.5
ChCl-QUE	N.O.	52.2	226.5	289.8	292.9

a N.O.: Not observed.

The viscosity of the NADES directly influences their ionic conductivity; therefore, viscosity is a crucial property to evaluate the suitabilities of specific NADES for several applications. As shown by the curves of viscosity vs shear rate presented in Figure S3, most NADES display nearly Newtonian behaviors in the range of 0.1–1000 s^–1^, except for ChCl-VA and ChCl-PGA, which exhibited an evident shear thinning behavior between 0.1 and 10 s^–1^. The viscosities of NADES increase in the following order: ChCl-PGA < ChCl-VA < ChCl-GEN < ChCl-HCA < ChCl-PHL < ChCl-CA ≪ ChCl-TA. The number of functionalities (such as −OH and −COOH) in the HBD seems to impact the viscosities of these green solvents greatly. In particular, multifunctional TA led to a highly viscous NADES, probably due to the establishment of multiple hydrogen-bonding interactions. On the other hand, the size of the polyphenols seem to play an essential role in the solvent viscosity. For instance, small-sized molecules like PGA and VA resulted in the two least viscous liquids, despite having two or more −OH groups, which can form strong hydrogen-bonding interactions. Similarly, ChCl-HCA showed higher viscosity than that of ChCl-GEN, despite having the same number of functional groups (two phenolic −OH and one −COOH). Another interesting behavior was found for ChCl-CA, which is much more viscous than ChCl-HCA, but the only difference between them is an unsaturation in their chemical structure. There is no clear explanation for this behavior, but we suppose that the double bond in CA allows for more molecular flexibility and freedom, promoting ion interactions and increasing viscosity.^11^

The viscosities of all NADES decreased when increasing the temperature, which can be correlated with the Arrhenius model, according to the following equation
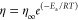
1where η is the viscosity in mPa s, η_∞_ a pre-exponential factor in mPa s, *E*_a_ the activation energy in kJ mol^–1^, *R* the ideal gas constant in kJ (mol K)^−1^, and *T* the temperature in Kelvin (K).

**Figure 4:**
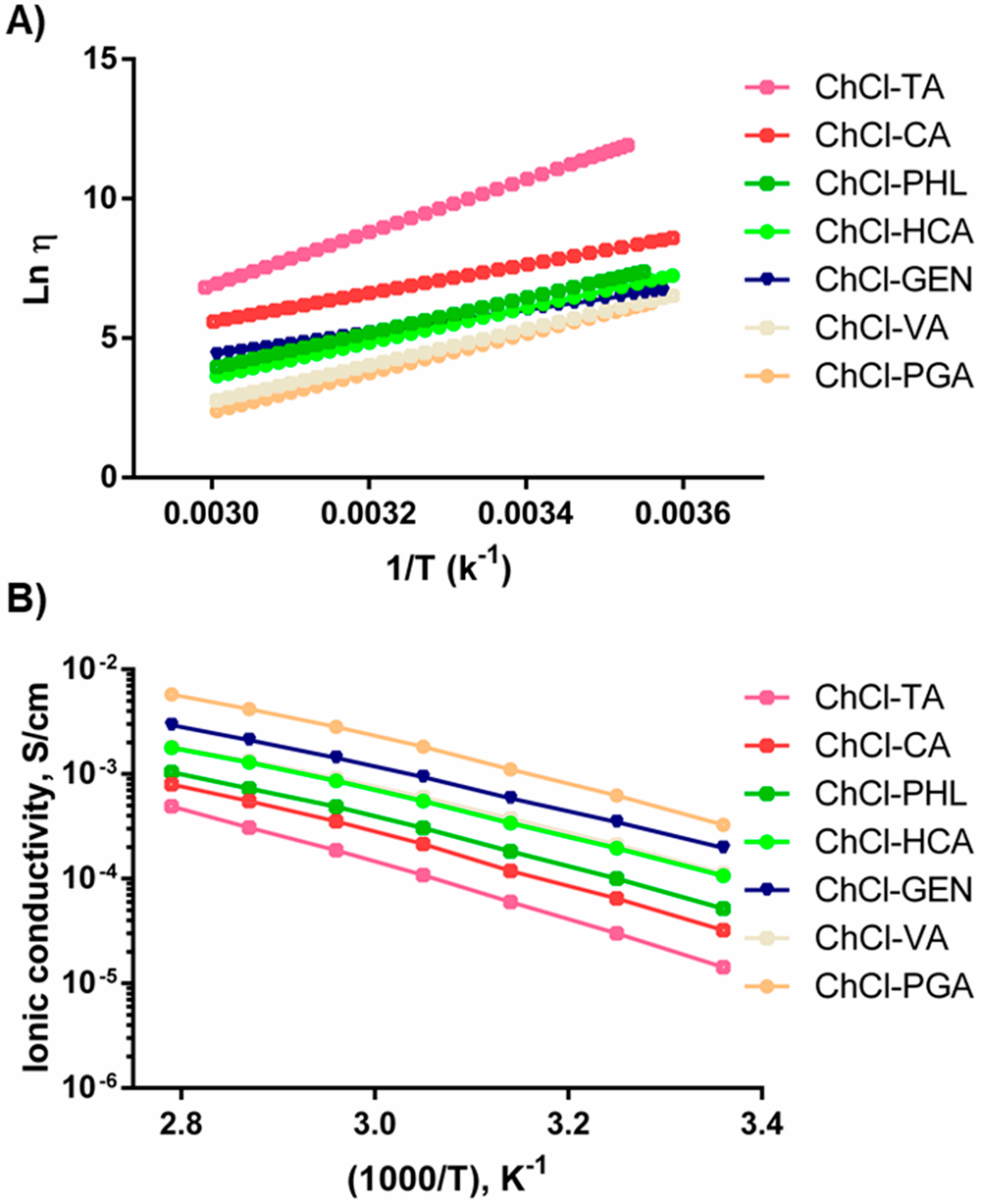
(A) Ln η vs 1/*T* plots derived from the Arrhenius model and (B) dependence of the ionic conductivity with temperature for the evaluated NADES.

The logarithmic form of eq 1 for the prepared NADES is plotted in Figure 4A. Note that the slopes of the plots give *E*_a_, which represents the activation energy barriers of NADES to shear stress. Specifically, the higher *E*_a_ is, the more complex the ions’ movements are, which is often associated with stronger interactions in the fluid lattice. The values of *E*_a_ obtained for each NADES are provided in Table S1 of the SI and varied as follows: ChCl-GEN < ChCl-CA < ChCl-HCA < ChCl-PHL < ChCl-VA < ChCl-PGA < ChCl-TA.

It should be noted that ChCl-PGA and ChCl-VA showed the second-highest *E*_a_ values despite their low viscosities. This behavior can be associated with the substantial decrease in the magnitude of hydrogen-bonding interactions with temperature. However, the highly viscous ChCl-CA revealed one of the lowest *E*_a_ values, supporting the idea that molecular associations, like π–π stacking, dominate the viscosity of this NADES.

The ionic conductivities of NADES and the influence of temperature on this key property were also investigated. As can be observed in Figure 4B, the NADES conductivities are closely related to their viscosities, showing an inverse relationship, as previously demonstrated for other NADES.^30^ It should be mentioned that ChCl-GEN is out of this trend, probably due to electrostatic interactions of the carbonyl group in the carboxylic acid structure, which contributes to the system polarization and overall ionic conductivity. The ionic conductivity values for the solvents at 25 °C ranged between 0.33 and 0.032 mS cm^–1^ for ChCl-PGA and ChCl-TA, respectively. These values are in the same order of magnitude as others previously reported for NADES based on ChCl-glycerol (1:2) and ChCl-oxalic acid (1:2).^1^ As expected, we also found that the ionic conductivities of the phenolic NADES are directly proportional to temperature due to the ions’ mobility enhancements.^31^

**Figure 5:**
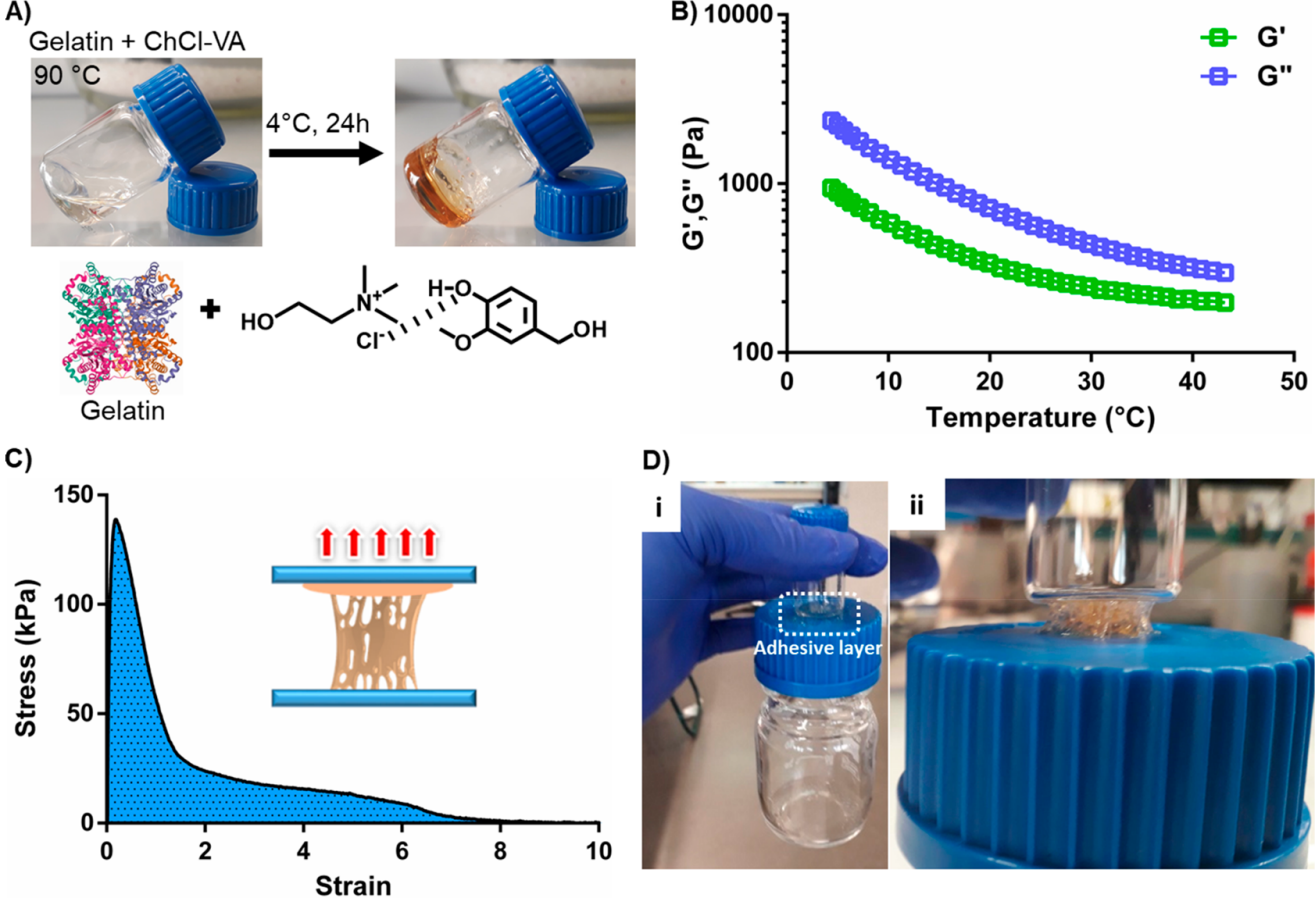
(A) Schematic representation of ChCl-VA/gelatin bioadhesive formation. (B) Temperature sweeps obtained by SAOS for the as-prepared adhesive material. (C) Adhesive stress vs strain for ChCl-VA/gelatin bioadhesive. (D) Photos of glass vials joint with ChCl-VA/gelatin bioadhesive (i) and adhesive fibrillation during debonding (ii).

Considering that ChCl-PGA and ChCl-VA NADES presented the lowest viscosities of the studied series, both liquids were combined with gelatin to prepare fully green soft ionic materials or gels. The use of DES in the preparation of ionic soft materials are a relatively recent research topic that has been attracting much attention in different applications.^32−36^ ChCl-PGA showed an excellent capacity to dissolve gelatin at room temperature. However, the mixture remained a viscous liquid after heating at 90 °C and cooling at 4 °C for 24 h, indicating that the gelatin’s triple helix formation was hindered in this NADES. On the other hand, the ChCl-VA/gelatin mixture resulted in a soft material after the heating/cooling process (Figure 5A), showing pretty interesting adhesive features.

Small amplitude oscillatory shear (SAOS) was performed to investigate the viscoelastic properties of the ChCl-VA/gelatin bioadhesive. The frequency sweeps revealed that ChCl-VA/gelatin behaves like a viscoelastic liquid with the viscous modulus (*G*′′) > elastic modulus (*G*′) in the range of 0.05–10 Hz (Figure S4A). This behavior was observed even at a very low strain of 0.01% (Figure S4B). In addition, temperature sweeps shown in Figure 5B revealed a softening of the adhesive material between 5 and 45 °C. Ultimately, we also evaluated the adhesive properties of the ChCl-VA/gelatin by a probe tack test (Figure S4C). As shown in Figure 5C, this material exhibited high adhesion energy of 310 J m^–2^ and an excellent tackiness of 136 kPa, which was much higher than that for Tisseel (≈20 kPa), a commercial fibrin tissue sealant, and comparable with other gelatin-based adhesives (≈110 kPa).^37^ As a proof of concept, we used the ChCl-VA/gelatin to join two vials, where an adhesive layer was applied between plastic and glass substrates. Image (i) of Figure 5D shows how the bioadhesive could firmly hold a big vial of around 160 g. Moreover, we found that the ChCl-VA/gelatin presents shear-thinning behavior (Figure S4D). Therefore, the potential of this ionic soft material as an injectable tissue bioadhesive could be further considered. It is worth mentioning that although polyphenols are substances generally recognized as safe (GRAS) by the U.S. Food and Drug Administration (FDA)^38^ and phenolic NADES are expected to be noncytotoxics, future biocompatibility tests are required to move forward in this application.

**Figure 6:**
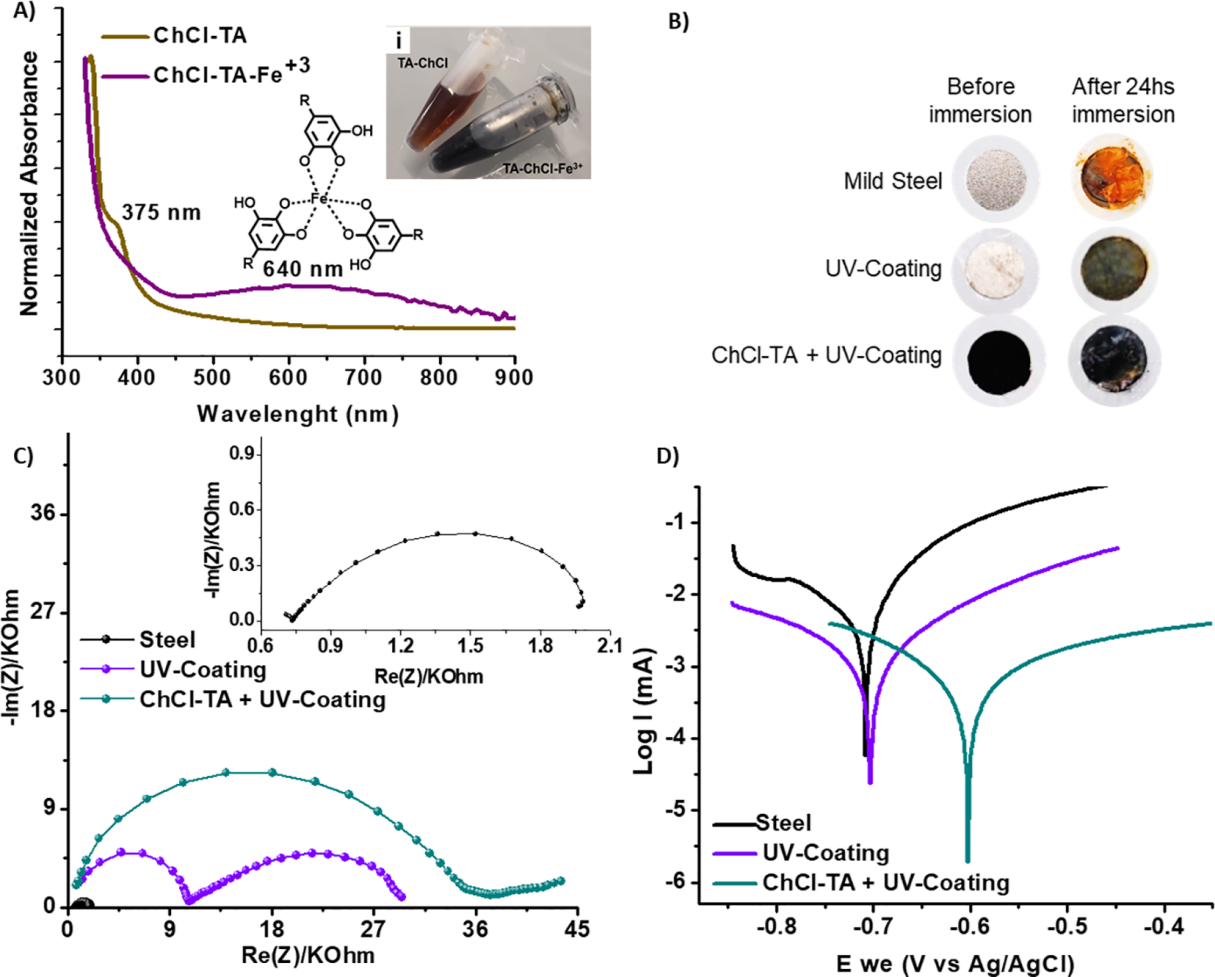
(A) UV spectra of ChCl-TA and ChCl-TA-Fe^3+^ complex. Inset: pictures of ChCl-TA and ChCl-TA-Fe^3+^. (B) Pictures of steel surfaces before and after 24 h of exposition to NaCl 0.01 M aqueous solution. Nyquist plot (C) and polarization curves (D) of samples after 24 h of immersion in a NaCl 0.01 M aqueous solution.

An attractive property of polyphenols is their outstanding ability as antioxidants and to coordinate metal ions. In particular, catechol and pyrogallol groups of TA have been exploited for engineering phenolic networks from various metals.^39^ Therefore, we explored the capability of ChCl-TA NADES to coordinate Fe^3+^ as a model metal ion. As shown in Figure 6A, ChCl-TA NADES has an absorption shoulder at 375 nm that corresponds to TA. However, upon adding FeCl_3_·H_2_O, a broad peak appeared at 640 nm, and the color of the NADES immediately turned dark red (Figure 6Ai), indicating the iron coordination to form a tris-complex state (see structure inset of Figure 6A).^40^

Tannic acid has been previously proposed for corrosion inhibition of ferrous metal objects in many environments.^41−43^ However, the ferric–tannates complex does not adhere well to metallic substrates and can be easily removed, providing a poor barrier effect.

The ability of ChCl-TA for iron coordination, together with its high viscosity, suggests that this NADES has a great potential to be applied as a corrosion inhibitor in mild steel AS1020 protection. Thus, we propose that ChCl-TA NADES can be used as a protection layer. ChCl-TA was combined with a UV-cured polymer coating to avoid the removal of the complexes formed on the steel surface and increase corrosion protection.

The corrosion tests were carried out using three samples: acrylic-coated mild steel, ChCl-TA + acrylic-coated mild steel, and cleaned mild steel AS1020 as a control (see Scheme 1 in Materials and Methods of the SI).

Electrochemical impedance spectroscopy measurements were carried out during immersion in a 0.01 M NaCl aqueous solution for 24 h. Pictures of the sample surface before and after this immersion period are presented in Figure 6B, while Figure 6C shows the corresponding Nyquist plots after 24 h of NaCl 0.01 M aqueous solution exposition. The sizes of the Nyquist capacitive semicircles are generally related to the degree of protection for each coated system. It can be seen that the application of ChCl-TA NADES in the coating showed the largest impedance, indicating a significant improvement in the corrosion protection of the steel. The impedance responses of the coatings and cleaned steel during 24 h are presented in Figure S5 as Bode’s plots. Interestingly, the coating formed with ChCl-TA presents the highest impedance values and a high phase angle value of 70°, showing the best anticorrosive profile.

The inhibitive efficiency (%η) and the corrosion parameters were obtained through Tafel extrapolation of the polarization curves (Figure 6D) and are provided in Table S2 of the SI. The inhibitive efficiency increased to around 93% when ChCl-TA NADES was applied on the steel surface, and it was similar to that of organic corrosion inhibitors, which are currently used in industrial applications.^44^ Besides, the ChCl-TA + acrylic coating shifts the corrosion potential (*E*_corr_) to more positive values, mainly due to the creation of a homogeneous barrier on the anodic sites upon the NADES formed TA–Fe^3+^ complexes, reducing the oxidation of Fe to Fe^2+^ when the surface is exposed to a corrosive environment (0.01 M NaCl aqueous solution.).

## Conclusions

Novel NADES were successfully prepared from a series of plant-derived polyphenols and choline chloride (ChCl). Although the common practice of testing mixtures with defined stoichiometric proportions was adopted to identify the new phenolic solvents, future research should address the building of solid–liquid equilibria phase diagrams to characterize these systems more deeply.

Most of the obtained NADES have a wide liquid range and high thermal stability above 150 °C. The results showed that the viscosities and ionic conductivities of NADES were strongly affected by the polyphenol structures. In particular, small phenolic molecules like pyrogallol (PGA) and vanillyl alcohol (VA) resulted in low-viscosity liquids (103 and 132 mPa s at room temperature) with high conductivities (1.1 and 3.3 × 10^–4^ S cm^–1^, respectively).

Furthermore, the potential of ChCl-VA NADES to produce bioadhesives in combination with gelatin was demonstrated, obtaining a soft material with better adhesive strength than a commercial formulation. On the other hand, the performance of ChCl-TA NADES as a corrosion protector of mild steel, in combination with an acrylic layer, was also tested. In this case, the formation of ChCl-TA-Fe^3+^ complexes on the steel surface plus a polymer coating increased the inhibitive efficiency up to 93% compared to 75% for the polymer coating alone.

Overall, the insights gained in this work will bring fresh perspectives to the preparation of novel polyphenol-based NADES.

## Supporting Information Available

Details on experimental methods and characterization, additional chemical structures scheme, NMR spectroscopy, NADES characterization data, corrosion parameters, and electrochemical impedance spectroscopy (PDF)
