# Supplementary material for: Natural Deep Eutectic Solvents Based on Choline Chloride and Phenolic Compounds as Efficient Bioadhesives and Corrosion Protectors
Source: ACS Sustain Chem Eng. 2022 Jun 13;10(25):8135–42. doi: 10.1021/acssuschemeng.2c01976 (PMC9241144; doi:10.1021/acssuschemeng.2c01976)
Supplement: Supplementary file 1 — sc2c01976_si_001.pdf [file sc2c01976_si_001.pdf]

Supporting Information (SI)

Natural Deep Eutectic Solvents Based on Choline Chloride and Phenolic Compounds as Efficient Bioadhesives and Corrosion Protectors

Matías L. Picchio,[a,b*†] Daniela Minudri,[c†] Daniele Mantione,[d] Miryam Criado-Gonzalez,[c] Gregorio Guzmán-González,[c] Ruth Schmarsow,[c] Alejandro J. Müller,[c,e] Liliana C. Tomé,[f] Roque J. Minari,[1] David Mecerreyes[c,e*]

[a]Instituto de Desarrollo Tecnológico para la Industria Química (INTEC), CONICET, Güemes 3450, Santa Fe 3000, Argentina.

[b]Departamento de Química Orgánica, Facultad de Ciencias Químicas (Universidad Nacional de Córdoba), IPQA−CONICET, Haya de la Torre y Medina Allende, Córdoba 5000, Argentina.

[c]POLYMAT and Department of Polymers and Advanced Materials: Physics, Chemistry and Technology, Faculty of Chemistry, University of the Basque Country UPV/EHU, Paseo Manuel de Lardizábal, 3, 20018 Donostia-San Sebastián, Spain.

[d]POLYKEY Polymers, Joxe Mari Korta Center, Avda. Tolosa 72, 20018 Donostia-San Sebastian, Spain.

[e] IKERBASQUE, Basque Foundation for Science, Plaza Euskadi 5, 48009, Bilbao, Spain.

[f]LAQV-REQUIMTE, Department of Chemistry, NOVA School of Science and Technology, FCT NOVA, Universidade Nova de Lisboa, 2829-516 Caparica, Portugal.

Corresponding authors:

Matías L. Picchio (E-mail: mlpicchio@santafe-conicet.gov.ar)

David Mecerreyes (E-mail: david.mecerreyes@ehu.es)

[†]These authors contributed equally to this work

Number of pages: 15

Number of figures: 5

Number of tables: 2

Experimental

Materials

All chemicals were used without any further purification. Choline chloride (ChCl, ≥98%), caffeic acid (CA, ≥98.0% HPLC), pyrogallol (PGA, ≥98.0% HPLC), tannic acid (TA, ACS reagent), gallic acid (GA, >97% titration) vanillyl alcohol (VA, 98%), hydrocaffeic acid (HCA, 98%), quercetin hydrate (QUE, ≥95%), vanillic acid (VAAc, 97%), 3,4-Dihydroxy-L-phenylalanine (L-DOPA, ≥98% TLC), gelatin from bovine skin (gel strength ~225 g Bloom, Type B), Iron(III) chloride hexahydrate (ACS reagent, 97%), 2-hydroxy-2-methylpropiophenone (97%), were purchased from Sigma-Aldrich. Gentisic acid (GEN, >98%), phloretic acid (PHL, >98%), protocatechuic acid (PCA, >98%), and trans p-coumaric acid (CUA, >98%) were purchased from TCI. Ellagic acid (97%) was purchased from Acros Organics. Dipropylene glycol diacrylate was obtained from Arkema/Sartomer. The chemical structures and respective acronyms of the NADES studied in this work are shown in Figure 1.

NADES preparation

NADES were prepared by the heating method, the most commonly used in the literature, based on mixing the two components (HBA and HBD) and heating them at 95°C under constant stirring until a homogeneous liquid is formed. The resulting mixtures were named ChCl-HBD.

ChCl-VA/gelatin adhesives were prepared by dissolving the protein (20% w/v) in the VA-based NADES at 90°C under constant stirring. Then, the solution was left at 4°C for 24 h to get a highly adhesive and viscoelastic liquid.

The ChCl-TA-Fe^{3+} complex was prepared by adding 180 mg of $FeCl_3{\cdot}6H_2O$ salt to 1 g of ChCl-TA and vortexed until dissolution. The TA: Fe^{3+} molar ratio

used was 1:3. The complex formation was then proved by UV-vis spectroscopy using an IMPLEN NanoPhotometer® NP80. Samples were measured without any dilution.

Characterizations

NMR spectroscopy

^{1}H and nuclear magnetic resonance (NMR) spectra were recorded in a Bruker Avance DPX 300 at 300.16 MHz, using deuterated dimethylsulfoxide (DMSO-d6) as solvent at room temperature.

FITR spectroscopy

Fourier transform infrared (FTIR) spectra were recorded on a Bruker Alpha II spectrophotometer employing a Platinum ATR module with a diamond window.

Thermal analysis

The thermal properties of the NADES were analyzed by thermogravimetric analysis (TGA) and differential scanning calorimetry (DSC). For TGA analysis, a TA Instruments Q500 equipment was used. Samples of about 30 mg were heated from 25 to 600 °C at a heating rate of 10 $°C \cdot min^{-1}$ and under a nitrogen atmosphere. The temperature at maximal decomposition rate (T_{max}) was determined as the temperature at the main peak of the derivative weight loss curve.

The DSC experiments were performed on a PerkinElmer 8500 DSC equipped with an Intracooler III. Samples of roughly 3 mg were crimped in non-recyclable aluminum hermetic pans and analyzed under a nitrogen atmosphere by heating and cooling cycles at a rate of 20 °C/min. First, the samples were heated from 25 to 100 °C and kept isothermally for 3 min to erase the thermal history.

Subsequently, the samples were cooled to –70 °C and kept isothermally for 10 min. Second-run heating cycles were conducted and further used to investigate the phase transition behavior of all samples.

Ionic conductivity measurements

Ionic conductivity (σ) was calculated from electrochemical impedance spectroscopy (EIS) measurements. A symmetrical stainless steel/NADES/stainless steel cell was assembled. The distance between the electrodes (l) was kept fixed at ~ 0.83 mm, using a silicone spacer ring with an inner area (A) of 0.502 cm^2. EIS measurements were carried out from 80 to 20 °C at every 10 °C intervals performed applying a 10 mV amplitude perturbation in the 1 MHz to 10 mHz frequency range at open circuit potential (OCP) conditions in a potentiostat galvanostat 302N from Autolab coupled to a Microcell HC temperature controller.

The fits of the experimental results were carried out using a nonlinear regression algorithm implemented in the Zview® software. The ohmic resistance (R_b) of the sample, estimated from the Nyquist plot at the low-frequency end of the semi-circle, was used to calculate σ using the following equation:

$$\sigma = \frac{1\ l}{R_b A} \qquad \text{(Eq. 1)}$$

Rheological measurements.

Rheological tests were carried out with an AR-G2 rheometer (TA instruments) using a cone-plate geometry of 20 mm radius, 1°59'36" angle, and 58 μm gap. First, the viscosity vs. shear rate, from 0.1 to 1000 s^{-1}, was measured at 25°C. Then, the temperature-dependent viscosities were measured from 4 to 100°C at

fixed shear rates of 1 s^{-1}, for high viscosity NADES, and 100 s^{-1} for low viscosity NADES.

For ChCl-VA/gelatin adhesives, a plate-plate geometry of 20 mm diameter and 500 µm gap was used. Oscillatory strain sweeps were carried out from 0.01 to 1000% strain at 1 Hz frequency and 25°C, and frequency sweeps from 0.01 to 40 Hz at 1% strain and 25°C. Temperature sweeps were performed from 4 to 45°C at 1% strain and 1 Hz frequency, and flow sweeps from 0.1 to 1000 s^{-1} at 25°C.

Probe-tack test

Adhesion measurements were performed using a TA HD plus Texture Analyzer equipment (Texture Technologies). Samples were prepared on glass support by applying an adhesive layer of about 1.5 mm thickness. In the probe-tack tests, a circular Delrin® probe (10 mm Ø) comes into contact with the sample at a given velocity of 1 mm/s. A 500 g compressive force is applied for 1 s, and the probe is removed from the film at a controlled velocity. The material tackiness was defined as the maximum stress, while the tack adhesion energy (W_{adh}) was calculated from the area under the stress-strain curve and the sample thickness.

Corrosion test

Preparation of mild steel and UV-polymerizable coating

Before the experiments, the mild steel AS1020 surface was cleaned and degreased with acetone at room temperature.

An acrylic UV-curable coating was composed of dipropylene glycol diacrylate and 2-hydroxy-2-methylpropiophenone (3 wt%) and applied onto the steel (or the steel and ChCl-TA) surface using a doctor blade technique, thus obtaining a

thickness coating of 200 μm in both cases (see Scheme S1). The precursor mixture was irradiated for 120 s using a UVC-5 (DYMAX) UV Curing Convenyor System with a lamp intensity of 400 mW·cm^{-2}.

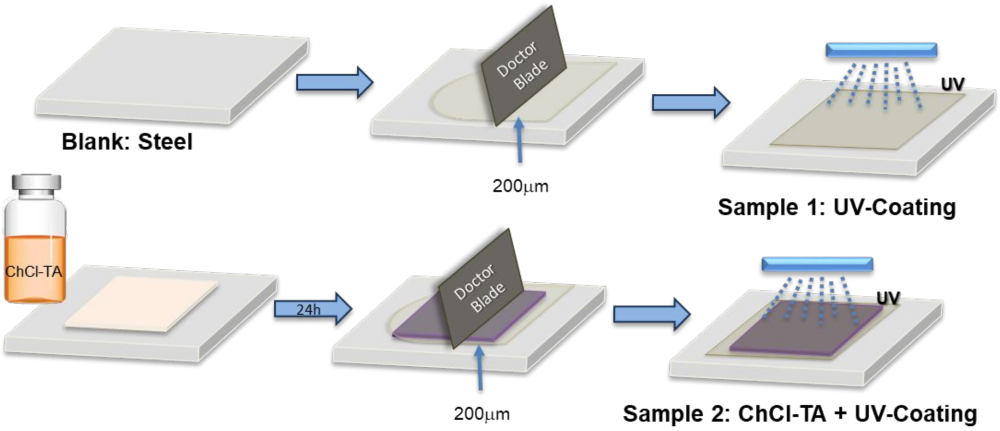

Scheme S1. Scheme of the coating layer preparation over steel using the doctor blade technique.

The acrylic coatings were formed by UV-curing of the monomer formulation (dipropylene glycol diacrylate + 2-hydroxy-2-methylpropiophenone) applied on the steel surface. For the ChCl-TA + polymer-coated samples, the steel was painted with ChCl-TA and left at room temperature for 24 hours to allow metal-iron coordination. When the surface was utterly dark red, an acrylic coating was formed in the same condition to compare the protective effect

Electrochemical measurements

A BioLogic VMP3 multi-channel potentiostat (Biologic, Seyssinet-Pariset, France) combined with EC Lab V10.44 software was used for the electrochemical experiments. A three-electrode cell was used with Mild Steel AS1020 as the working electrode, graphite rod as the counter electrode, and Ag/AgCl (3M KCl) as the reference electrode. Samples were immersed in 0.01M NaCl aqueous solution for 24 hours, and the immersed area was 0.95 cm^2.

Potentiodynamic polarization measurements

Potentiodynamic polarization curves were obtained by changing the potential from -150 to +250 V vs. open circuit potential (OCP) with a scan rate of 2 mV· s^{-1}. We use the Tafel extrapolation to obtain the corrosion parameters, and the inhibitive efficiency (η%) was calculated through the following expression.

$$\eta\% = \frac{I^0_{corr} - I_{corr}}{I^0_{corr}} x100$$

Where I^0_{corr} and I_{corr} are the corrosion current densities without and with inhibitor coatings, respectively.

Electrochemical Impedance Spectroscopy (EIS)

Electrochemical impedance spectroscopy measurements were carried out using the aforementioned BioLogic VMP3 potentiostat. The the impedance at the OCP was monitored in the frequency range from 1 MHz to 10 mHz, with 6 points per decade and a sinusoidal amplitude of 10 mV. Impedances responses were monitored after each hour during 24 h of exposition to 0,01M NaCl solution.

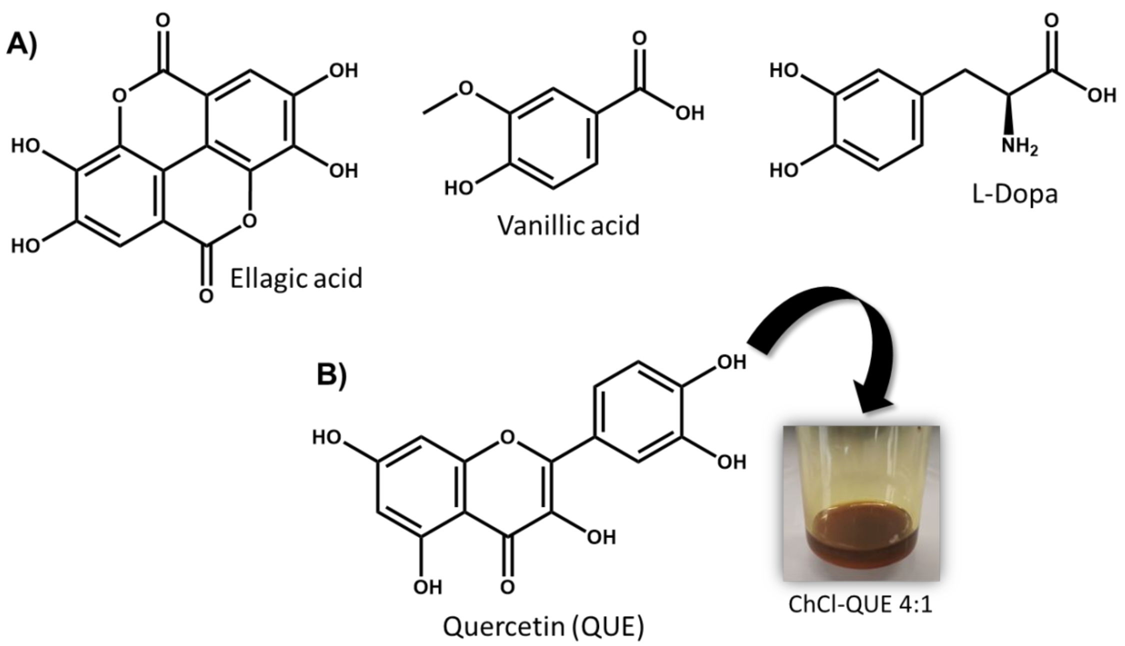

Figure S1. Chemical structures of (A) different polyphenols that showed no interaction with ChCl and (B) the flavonol QUE yielding a NADES at 4:1 molar ratio.

ChCl-PHL 1:1

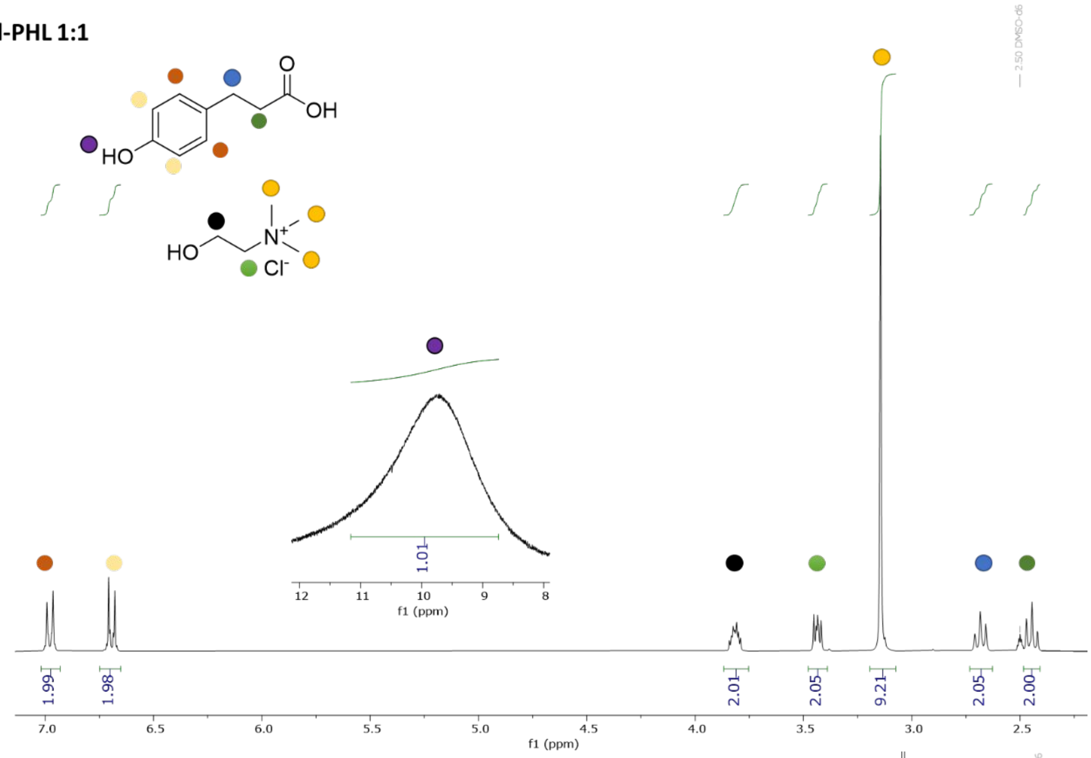

ChCl-GEN 2:1

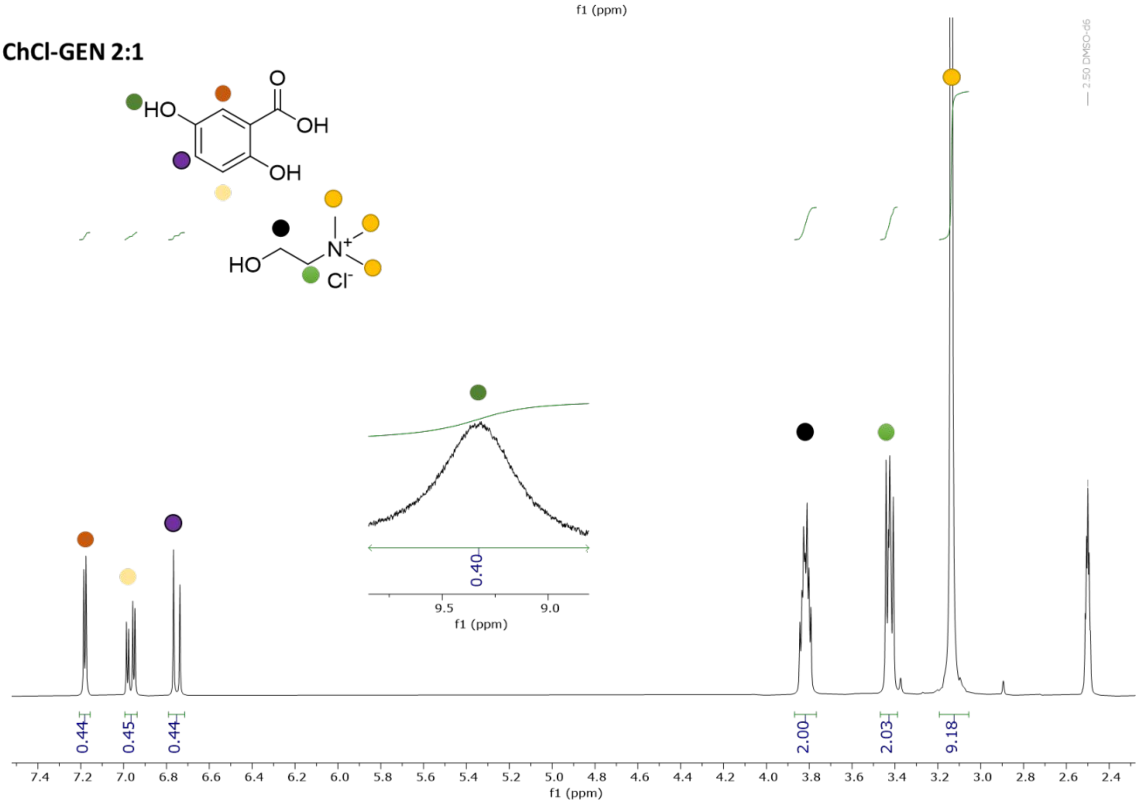

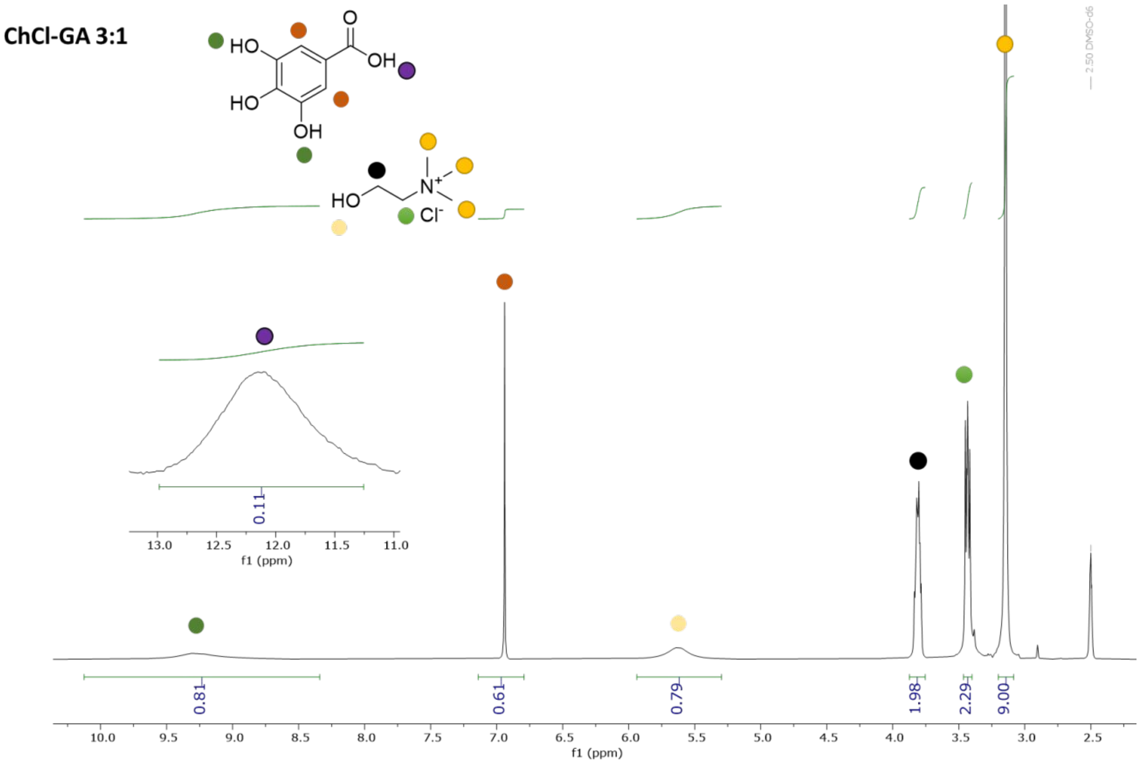
ChCl-GA 3:1
HO
HO
OH
O
OH
HO
N+
Cl-
0.11
13.0
12.5
12.0
11.5
11.0
f1 (ppm)
0.81
0.61
0.79
1.98
2.29
9.00
10.0
9.5
9.0
8.5
8.0
7.5
7.0
6.5
6.0
5.5
5.0
4.5
4.0
3.5
3.0
2.5
f1 (ppm)

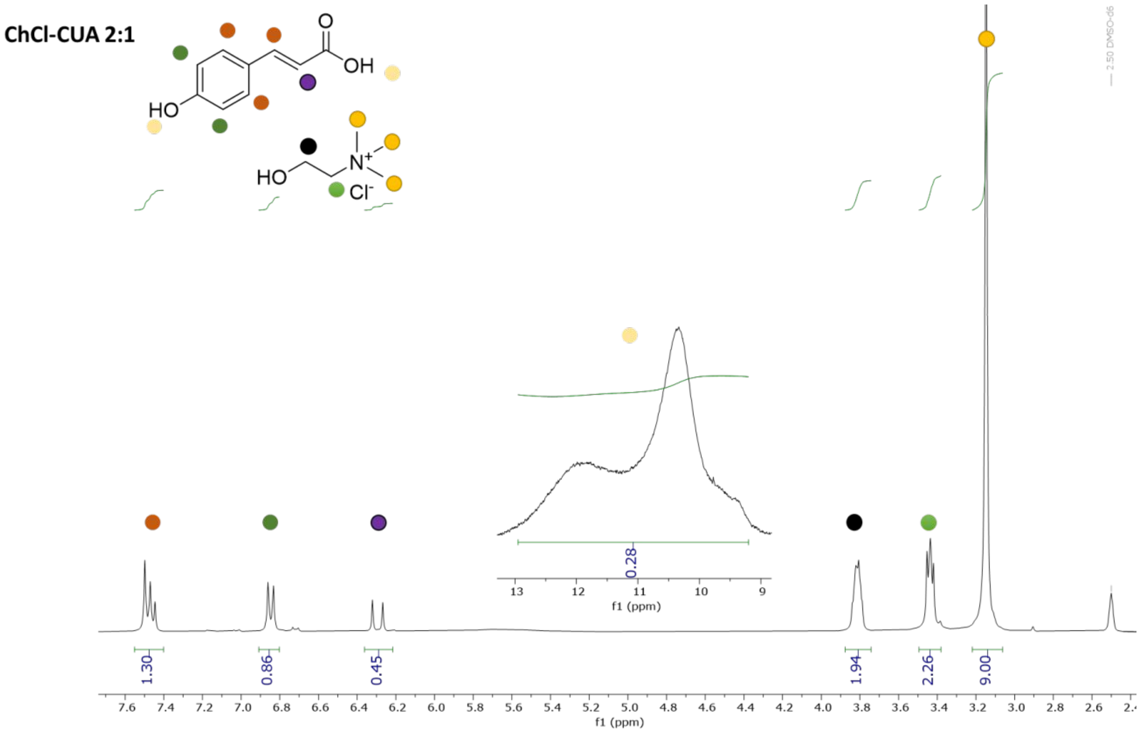
ChCl-CUA 2:1
O
OH
HO
HO
N+
Cl-
0.28
13
12
11
10
9
f1 (ppm)
1.30
0.86
0.45
1.94
2.26
9.00
7.6
7.4
7.2
7.0
6.8
6.6
6.4
6.2
6.0
5.8
5.6
5.4
5.2
5.0
4.8
4.6
4.4
4.2
4.0
3.8
3.6
3.4
3.2
3.0
2.8
2.6
f1 (ppm)

ChCl-PGA 1:1

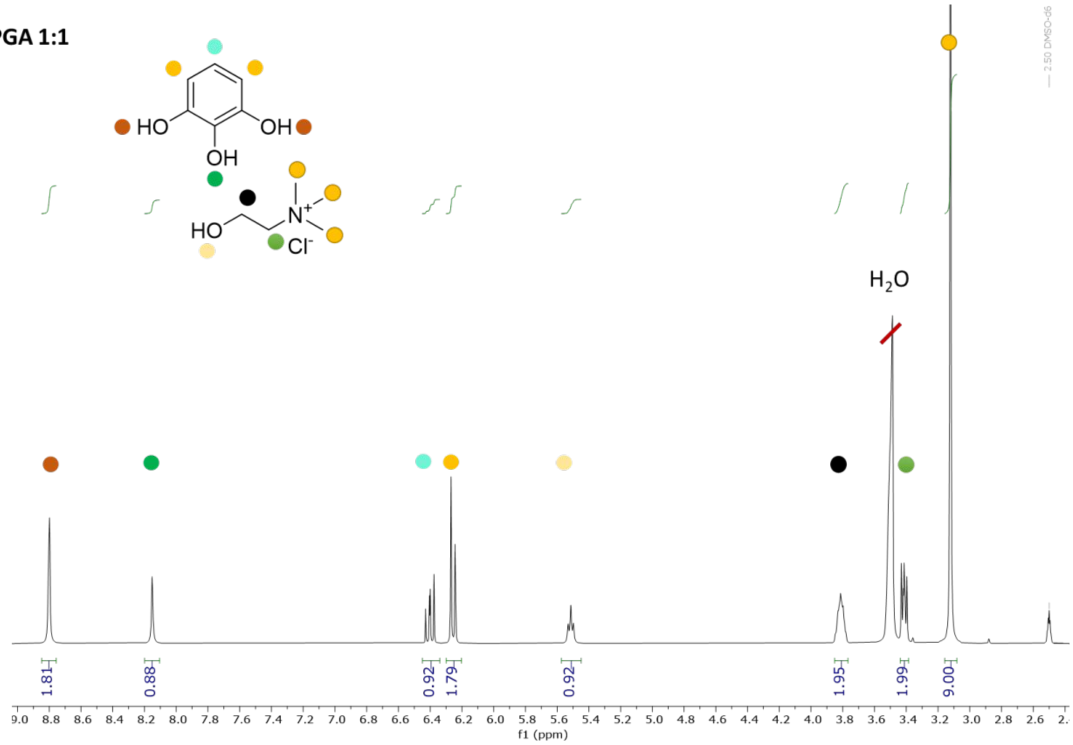

ChCl-PCA 2:1

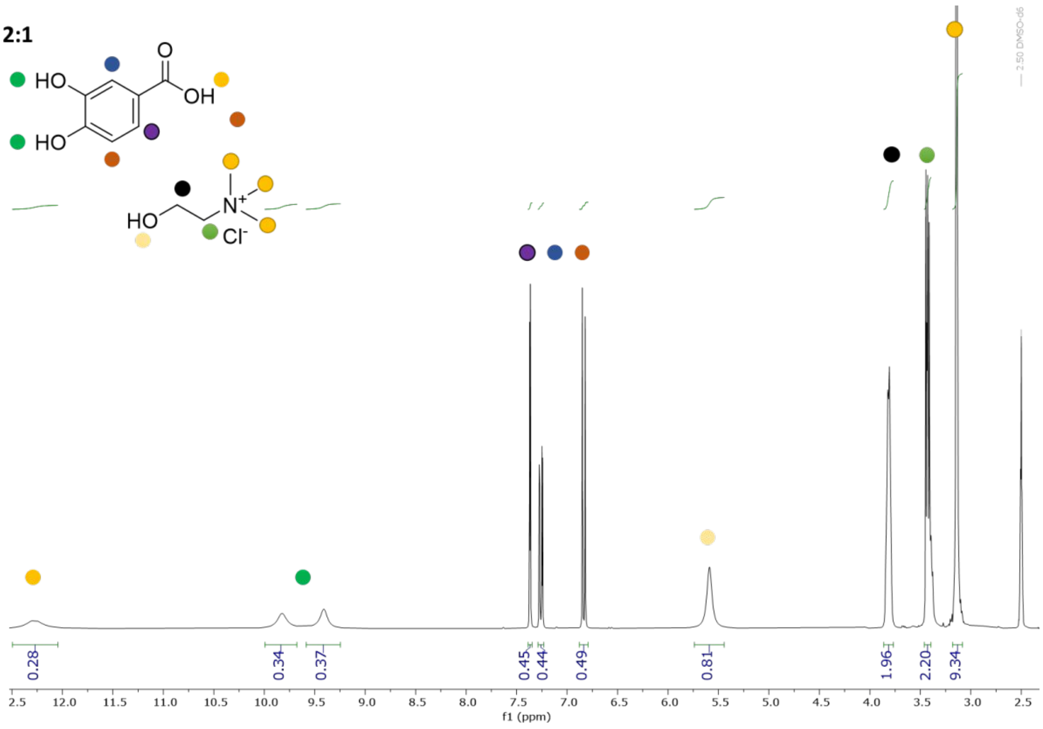

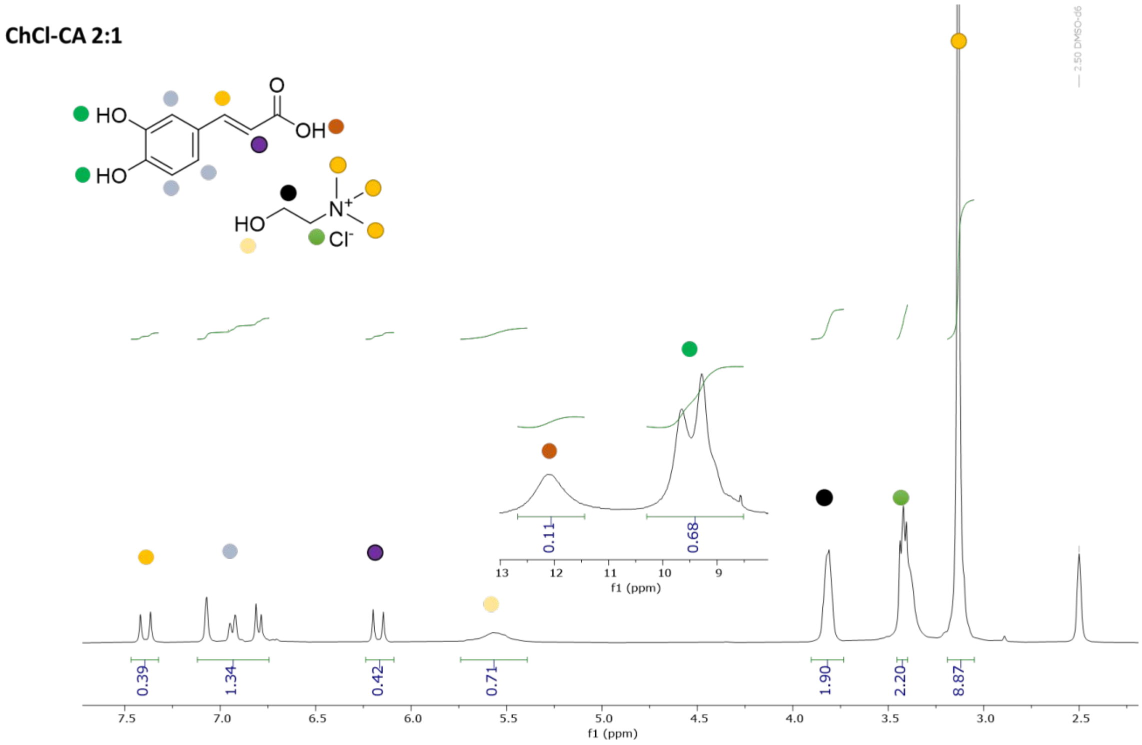

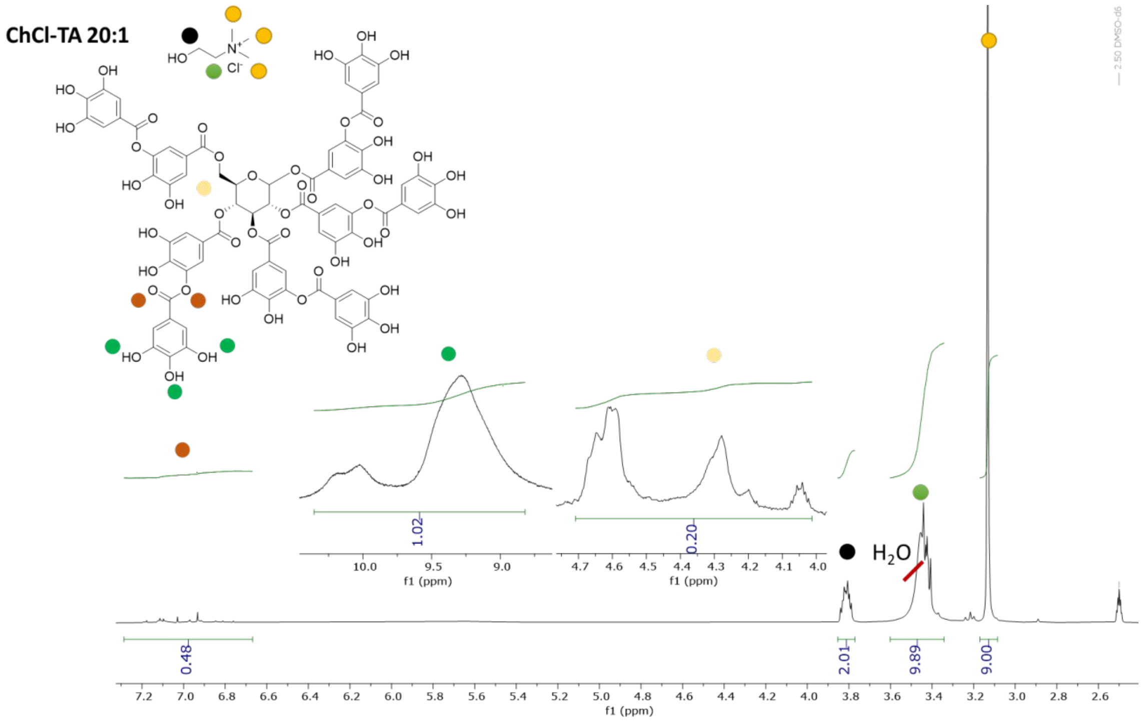

Figure S2. H[1] NMR spectra of the NADES formed by ChCl and different natural phenolic compounds.

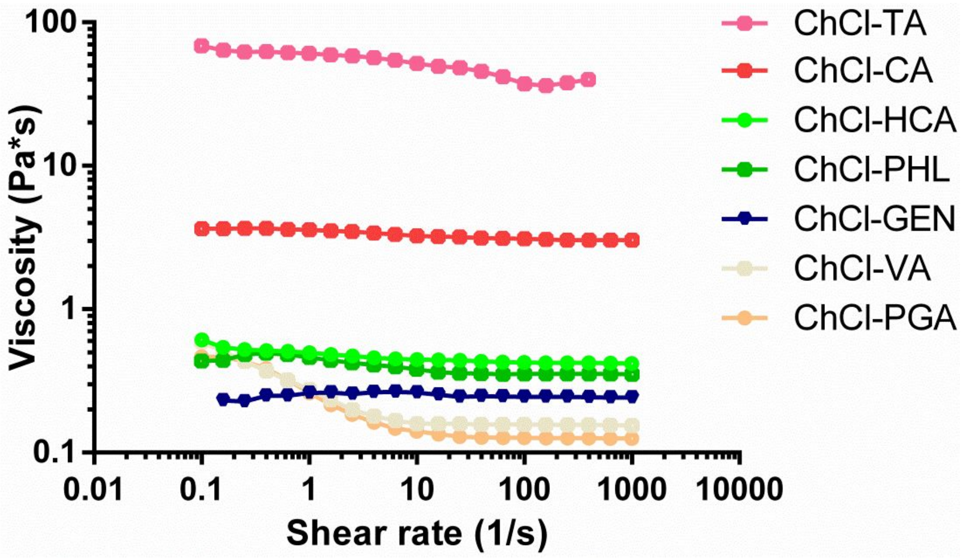

Figure S3. Viscosity *vs.* shear rate curves for the polyphenols-based NADES.

Table S1. E_a values for the viscosity of NADES as a function of temperature, determined by Eq 1.

NADES	E_a (kJ/mol)	R^2
ChCl-TA	9493.3	0.9951
ChCl-PGA	6997.1	0.9844
ChCl-VA	6474.3	0.9728
ChCl-PHL	6296.7	0.986
ChCl-HCA	6201.3	0.9971
ChCl-CA	5121.3	0.9856
ChCl-GEN	4043.9	0.989

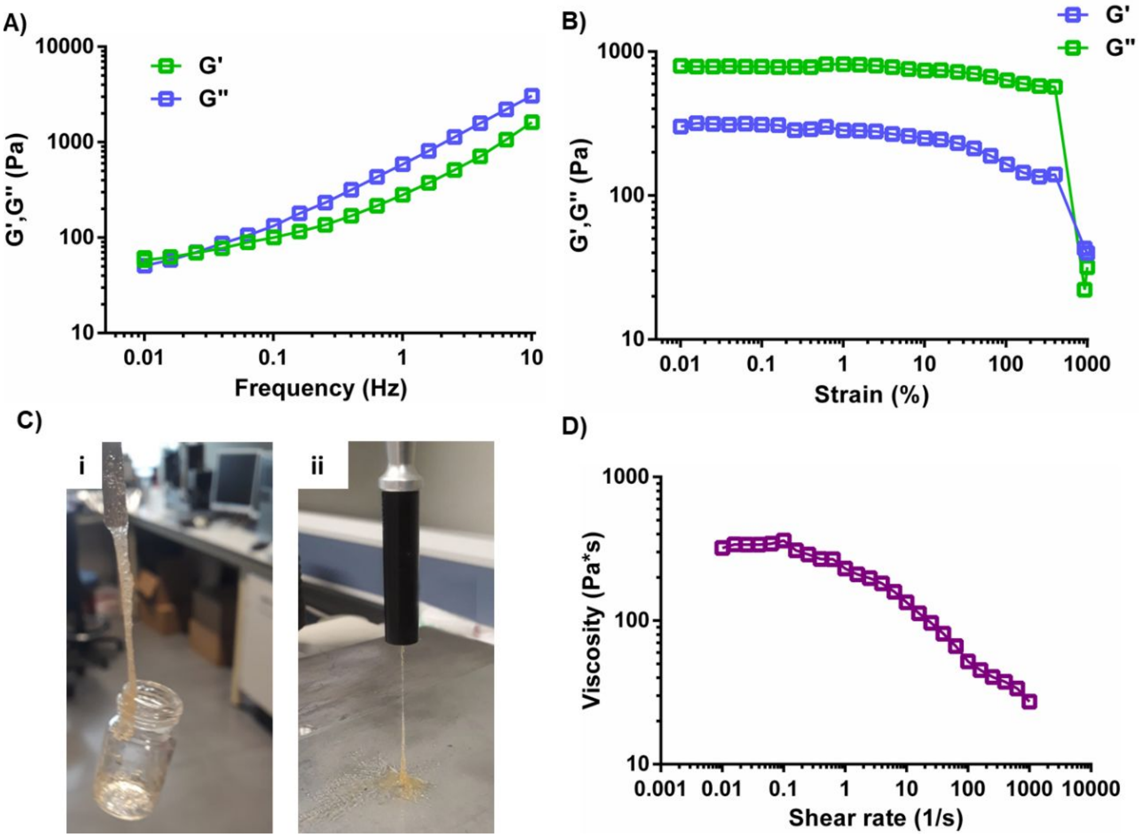

Figure S4. (A) Frequency and (B) amplitude sweeps for ChCl-VA/gelatin bioadhesive at 25 °C and 1 Hz. (C) Pictures of: one glass vial hanging from the bioadhesive material (i); and adhesive debonding during probe tack test (ii). (D) Flow curve for the adhesive material at 25 °C.

Table S2. Corrosion parameters and inhibition efficiency obtained from polarization curves after 24 h immersion in 0,01M NaCl aqueous solution.

	**- E_{corr}** (V vs Ag/AgCl)	**I_{corr}** ($\mu A\ cm^{-2}$)	**βa** (mV/dec)	**Bc** (mV/dec)	**%η**
Steel	0.707	21.3	16.7	24.8	-
UV-Coating	0.704	5.4	19.1	23.1	74.6%
ChCl-TA+ UV-Coating	0.602	1.4	21.5	21.5	93.4%

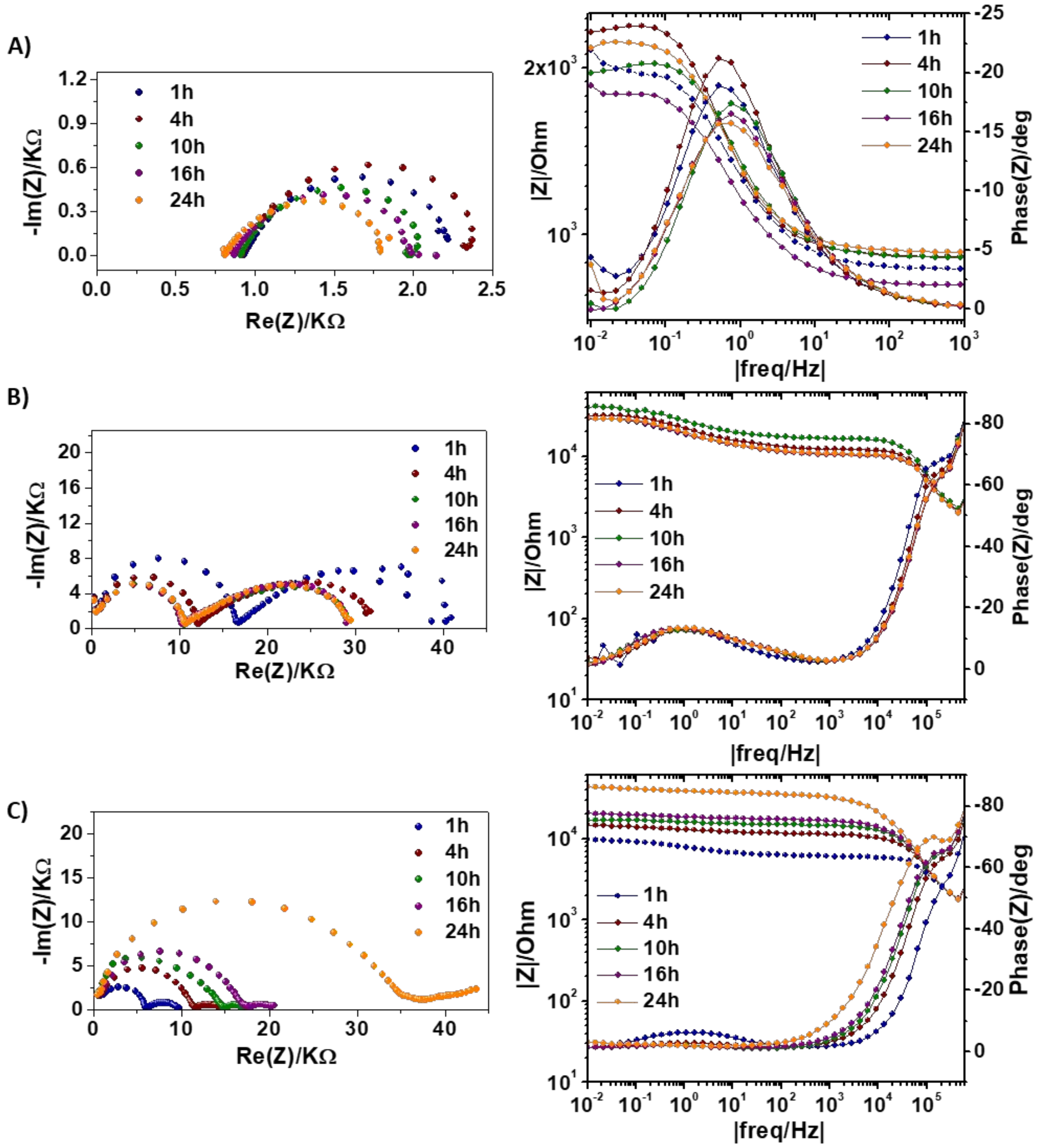

Figure S5: Nyquist plots and Bode plots of (A) cleaned mild steel, (B) acrylic-coated mild steel and (C) ChCl-TA + acrylic-coated mild steel along the immersion time.
